# Carbon Black as Conductive Additive and Structural Director of Porous Carbon Gels

**DOI:** 10.3390/ma13010217

**Published:** 2020-01-04

**Authors:** Ana Casanova, Alicia Gomis-Berenguer, Aurelien Canizares, Patrick Simon, Dolores Calzada, Conchi O. Ania

**Affiliations:** 1 CEMHTI, CNRS (UPR 3079), University Orléans, 45071 Orléans, France; ana.casanova-martinez@cnrs-orleans.fr (A.C.); alicia.gomis-berenguer@cnrs-orleans.fr (A.G.-B.); aurelien.canizares@cnrs-orleans.fr (A.C.); patrick.simon@cnrs-orleans.fr (P.S.); 2 Laboratorio de Innovación en Plasmas (LIPs), Universidad de Córdoba, 14071 Córdoba, Spain; md.calzada@uco.es

**Keywords:** nanoporous carbon gels, conductive additives, carbon black, electrical conductivity, percolation, mesoporosity, rule of mixtures

## Abstract

This work investigates the impact of carbon black (CB) as a porogenic agent and conductive additive in the preparation of electrically conductive nanoporous carbon gels. For this, a series of materials were prepared by the polycondensation of resorcinol/formaldehyde mixtures in the presence of increasing amounts of carbon black. The conductivity of the carbon gel/CB composites increased considerably with the amount of CB, indicating a good dispersion of the additive within the carbon matrix. A percolation threshold of ca. 8 wt.% of conductive additive was found to achieve an adequate “point to point” conductive network. This value is higher than that reported for other additives, owing to the synthetic route chosen, as the additive was incorporated in the reactant’s mixture (pre-synthesis) rather than in the formulation of the electrodes ink (post-synthesis). The CB strongly influenced the development of the porous architecture of the gels that exhibited a multimodal mesopore structure comprised of two distinct pore networks. The microporosity and the primary mesopore structure remained rather unchanged. On the contrary, a secondary network of mesopores was formed in the presence of the additive. Furthermore, the average mesopore size and the volume of the secondary network increased with the amount of CB.

## 1. Introduction

Nanoporous carbons are key materials in many electrochemical applications over a wide variety of competitors (such as noble metals, non-noble metals, and metal oxides) due to the diversity of carbons with controlled pore architectures combined with adequate bulk and surface properties; particularly, chemical and mechanical stability, biocompatibility, rich surface chemistry, and, most importantly, relatively high electronic conductivity [1,2].

Although some carbons (e.g., graphite, graphene and its derivatives, carbon nanotubes) present electronic properties close to those of metallic electrodes, this feature depends strongly on the spatial arrangement of the carbon atoms. Indeed, most nanoporous carbons are non-polycrystalline materials with a low degree of structural order, as a result of a high density of defects introduced in the twisted graphitic layers upon the development of a nanopore network. As a result, the electron mobility pathway characteristic of the graphenic sheets is greatly reduced, limiting the conductivity of nanoporous carbons (typically 4–5 orders of magnitude lower than that of graphite or graphene) [2,3]. To increase the conductivity of nanoporous carbon electrodes without compromising the nanoporosity, several strategies have been explored, such as: (i) doping the carbon material with heteroatoms [4]; (ii) coating with a conductive phase, such as metallic nanoparticles, metals, or other conductive carbon nanostructures [5,6]; (iii) synthesizing 3D nanoporous graphene-like architectures [7]; and (iv) incorporating a conductive additive in the formulation of carbon electrodes inks [1,3,8]. The latter is the common practice for the manufacturing of the electrodes on a commercial scale in most electrochemical applications, carbon black (CB) being the most popular conductive additive due to its low cost, and reasonably high chemical stability and electrical conductivity.

However, the optimum content of a conductive filler needed to achieve an increase in the electrical conductivity (known as the percolation threshold) through an adequate “point to point” conductive network [9] is quite high for CB. This is due to the morphology of the CB nanoparticles (typically globular aggregates with diameters of a few tens of nanometers). Indeed, the development of a uniform conducting architecture is essential to enhance the conductivity of the final composite, and the characteristics of the additive (type, particle size and shape, orientation in the matrix) play a key role in revealing percolation properties. As a result, the electrochemical response of the electrodes prepared with CB-based ink formulations is limited [8,9,10]. Aiming at lowering the percolation thresholds with a minimal impact on the electrochemical response of the carbon electrodes, some other carbon nanostructures with high electrical conductivities and aspect ratios (such as carbon nanotubes, carbon nanofibers, and graphene derivatives) have been considered to replace CB as the conductive additive [11,12,13,14]. Despite the promising decrease in percolation threshold, the cost of these carbon materials is still too high to make them competitive with carbon black as conductive additives in large scale applications.

In a previous study we explored a different approach, consisting of incorporating the carbon black additive during the synthesis of the nanoporous carbon material itself, as opposed to its incorporation in the electrode ink formulation [15]. The choice of the carbon electrode was thus dictated by a synthesis route allowing the incorporation of the additive in a pre-synthesis step; within this context we selected carbon aerogels prepared by the polycondensation of resorcinol and formaldehyde mixtures, since it is possible to obtain highly porous materials with tunable properties while modifying the synthesis process to incorporate the conductive additive [15,16]. Indeed, after the early works of Pekala and co-workers reporting their preparation [17], nanoporous carbons gels have become interesting materials with an outstanding performance in various fields as adsorbents, catalyst supports, energy storage devices, and electrochemistry [18,19,20]. Our studies showed that the incorporation of low amounts of carbon black during the polycondensation of the reactants allowed their polymerization and cross-linking, leading to the preparation of highly nanoporous carbon gels with improved conductivity, and thus electrochemical performance.

In view of the above, the present study aimed to evaluate the percolation threshold of carbon black as the conductive additive incorporated during the synthesis of nanoporous organic and carbon gels. These materials were prepared following the sol-gel polycondensation reaction of resorcinol/formaldehyde mixtures in the presence of increasing amounts of carbon black. The characteristics of the resulting carbon materials were evaluated upon the amount of conductive additive. The percolation threshold of the carbon black additive on the structure, porosity, and electrical conductivity of the nanoporous carbon gels was analyzed based on experimental measurements and analytical models.

## 2. Materials and Methods

### 2.1. Synthesis of Materials

Hydrogels were synthesized by the polycondensation of resorcinol (R, 99% purity, Sigma Aldrich, St. Louis, MO, USA) and formaldehyde (F, 37 wt.% in water, stabilized by 10%–15 wt.% of methanol) in water (W) using sodium carbonate as catalyst (C, 99% purity, Sigma Aldrich, St. Louis, MO, USA), carbon black (CB, Superior Graphite Co., Chicago, Illinois, USA) as conductive additive, as indicated elsewhere [15,16]. In a typical synthesis, the precursors (molar ratio R/F 0.5, R/C 200 and R/W, 0.06) were mixed and transferred to airtight sealed glass vessels for gelation/aging at 70 °C for 4 h using an oil bath, followed by drying at 150 °C for 12 h in an oven without solvent removal. The carbon black additive was incorporated to the resorcinol solution and sonicated (ca. 15–30 min, FisherBrand 112, Thermo Electron SAS, Villebon Courtaboeuf, France). The ratios of carbon black varied between 0 and 40 wt.% expressed as grams of CB per grams of reactants (R + F). Subsequently, the formaldehyde solution was added to the resorcinol/carbon black dispersion, and a mechanical stirring (ca. 500 rpm) was maintained during the gelation step at 70 °C. When the gel started to be formed (ca. after 2 h of gelation), and thus the density of the precursor’s mixture increased, the stirring was naturally stopped. The organic gels were labeled as PG-CBZ, where Z accounts for the amount of carbon black additive. After drying at 150 °C, the gels were grinded in a ball milling (PM 100 Retsh, Haan, Germany) and carbonized at 800 °C under a nitrogen flow of 120 mL/min in a horizontal tubular furnace (HST, Carbolite Gero, Hope Valley, UK) (heating rate of 2 °C/min with 3 dwelling steps of 60 min at 200, 400, and 800 °C). The nomenclature of the carbonized gels is CPG-CBZ.

### 2.2. Characterization Techniques

The porosity of the materials was evaluated by gas adsorption isotherms (e.g., N_2_ and CO_2_ at −196 and 0 °C, respectively) in automatic volumetric analyzers (Micromeritics, Norcross, GA, USA). The samples were initially degassed under vacuum at 120 °C for 17 h. The nitrogen adsorption isotherms were used to calculate the specific surface area (S_BET_), total pore volume (V_PORES_), micropore volume (W_0_, using the Dubinin–Radushkevich (DR) equation) [21], and pore size distribution (PSD) using the 2D-NLDFT-HS model for carbons with surface heterogeneity [22]. The narrow microporosity was further assessed from the CO_2_ adsorption isotherms using the Dubinin–Radushkevich equation. Each isotherm was recorded in duplicate on fresh sample aliquots, to guarantee the accuracy and reproducibility of the experiments (error was below 2%). The nanostructures of the materials were characterized by transmission electron microscopy (TEM) using a microscope (Philips CM20, Philips Co. Ltd., Amsterdam, Holland) operating at 200 kV. Powder samples were dispersed in ethanol and deposited on a holey carbon film supported by a copper grid. Powder X-ray diffractograms were recorded in a Bruker diffractometer (D8 Advance, Manning Park, Billerica, MA, USA) operating at 30 kV and 40 mA and using CuKα (0.15406 nm) radiation. Data were collected between 5° and 90° with a 0.08° step size. Fourier-transform infrared (FTIR) spectroscopy studies were carried out with a Bruker Vertex 80 v (Billerica, MA, USA) using materials dispersed in and pressed with dry KBr, keeping a 1:100 ratio (w/w). Transmission spectra were carried out between 4000–350 cm^−1^ (64 scans collected, resolution 4 cm^−1^).

Raman spectra (Renishaw InVia Qontor, Renishaw SAS, Marne la Vallée, France) were recorded in ambient conditions in a spectrometer equipped with 514.5 nm laser. The spectra were collected under a Leica DM2500 optical microscope with a ×50 long working distance objective (ca. 10 mm). The scattered Raman light was dispersed by a holographic grating of 600 grooves/mm, in order to acquire the whole range of interest for carbons (500–5000 cm^−1^). Each spectrum was recorded with an integration time of 5 s; data presented represent the averages of three measurements. Raman imaging was performed using the fast Streamline mode of the spectrometer setup. A zone of ca. 180 × 115 µm^2^ on the samples was scanned with a step of 1.3 μm in two dimensions (accounting for the spatial resolution), resulting in 12,000 acquired spectra recorded over 19.5 h of experimentation. The LiveTrack^TM^ mode was used, allowing us to maintain the focus automatically during the measurements whatever the surface state, which becomes essential when recording powders. Wire^TM^ software (v4, Renishaw SAS, Marne la Vallée, France) was used to remove cosmic rays, and to perform the spectral curve fitting (line positions are obtained by a classical Gaussian/Lorentzian fitting process).

The electrical conductivity of the samples was measured using a four-point probe method following the general principles of ASTM standard methods D4496-87 [23]. Briefly, pellets of the samples (ca. 90 wt.% of carbon powders, 10 wt.% polyvinylidene fluoride binder) were prepared by compaction of the powders under 5 tons pressure. The diameter of each pellet was ca. 10 mm and their thicknesses varied between 0.10 and 0.16 mm (total weights between 8–12 mg). Resistance of the pellets was measured at room temperature and atmospheric pressure using a Lucas Labs four-point probe stand (S-302-6) with a Signatone four point probe head (SP4-62-045-TBY) to make electrical contact. A constant current (between 0.1 and 10 mA) was applied to the surface of the pellets through the probes, and the voltage drop was recorded. The bulk resistivity of the samples (ρ, Ω∙m), reciprocal of conductivity (σ) was calculated according to

(1) $$\rho \;=\;\frac{U}{I}\frac{\pi }{\ln2}t$$

where U is the voltage drop (V), I is the current intensity (A), and t is the thickness of the pellets (m) [24].

## 3. Results and Discussion

### 3.1. Synthesis of the Materials

In a previous study, we reported the successful polycondensation of resorcinol-formaldehyde mixtures in the presence of low amounts of additives (e.g., diatomite, carbon black), to render carbon gel/CB composites with enhanced electrical conductivity and mechanical features [25,26]. Aiming to evaluate the percolation threshold of carbon black in its role as conductive additive, we prepared a series of organic and carbon gels with a fixed molar ratio of reactants (R/F, R/C and R/W) and increasing amounts of CB additive.

Due to its hydrophobic nature, the CB was initially dispersed in the resorcinol solution by sonication 15–30 min before adding the formaldehyde and the catalyst. The suspension with all the reactants was maintained under mechanical stirring to avoid the sedimentation of the CB nanoparticles during the different steps of the synthesis (e.g., polycondensation and gelation). This step became critical for those materials prepared with high amounts of carbon black (ca. above 16 wt.%) so as to assure a homogeneous distribution of the CB nanoparticles in the resulting gels.

It is important to mention that the gelation of the reactants occurred within a similar timescale for all the samples, regardless the presence of the CB additive and upon mechanical stirring. This indicates that neither the CB additive nor the stirring affect the stiffing of the sol-gel characteristic of the polymerization of R/F mixtures [18,20]. Indeed, a heterogeneous distribution of the CB in the gels was obtained when the dispersions were not stirred before the gelation step, as a consequence of the sedimentation and accumulation of CB nanoparticles in the bottom of the reaction vessel (Figure S1 in the Supplementary Materials). It should also be pointed out that the mechanical stirring alone did not affect the porosity of the gel, as corroborated by gas adsorption analysis (Figure S1).

**Figure 1:**
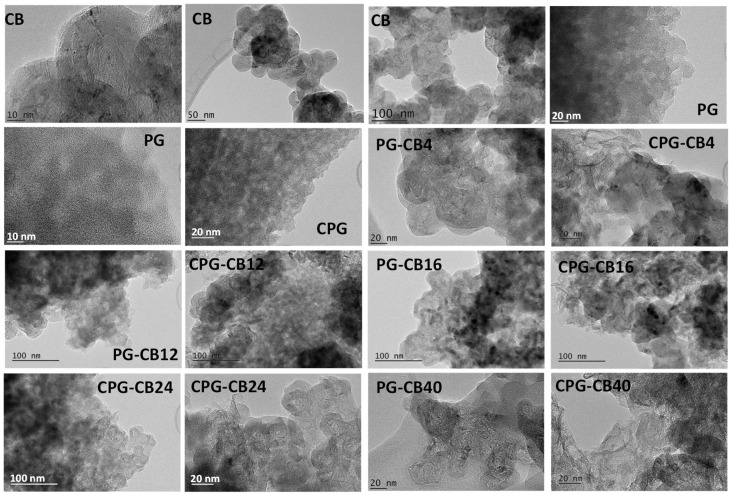
Selected TEM images of the carbon black additive, and the organic (series PG) and carbon gels (series CPG) prepared with different amounts of carbon black. For clarity, images are shown at various magnifications.

Figure 1 shows the TEM images of the gels and the carbons prepared in the absence and the presence of the different amounts of CB additive. Images of the carbon black are also included as references, showing the spherical-shaped nanometric aggregates (low aspect ratio, ca. 20 nm diameter) with the characteristic graphitic domains of conductive carbon black. For the samples prepared in the absence of CB—samples PG and CPG—the wormhole-like characteristic fingerprint of a disorganized matrix was observed. In the presence of CB, the spherical-shaped nanometric aggregates of the conductive additive were clearly distinguished within the matrix of the organic and the carbon gels. Even those materials prepared with high CB content displayed a continuous conductive network of the CB aggregates. This was expected to have a beneficial impact in the electronic conductivity of the samples (see discussion below).

As mentioned above, the uniform distribution was guaranteed by the mechanical stirring in the initial steps of the synthesis that prevented the sedimentation of the conductive additive (Figure S1). No significant differences were observed in the distribution and/or length of the conductive networks of the organic gels (series PG, before pyrolysis) compared to those in the corresponding carbon gels (series CPG, after carbonization). This is interesting, since during carbonization a large fraction of volatiles is removed (around 40–50 wt.%), and indicates that there are not structural rearrangements between the organic matrix of the gel and the CB additive during carbonization, that might otherwise favor the aggregation of the CB in the carbon gels.

### 3.2. Structural Characterization

**Figure 2:**
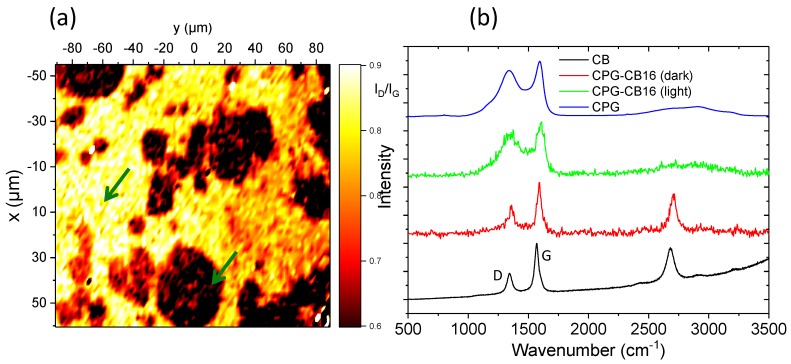
(**a**) Raman image reconstruction showing the I_D_/I_G_ ratios for samples CPG–CB16; (**b**) Raman spectra of CB, CPG, and two different positions in the reconstructed Raman mapping for sample CPG–CB16 corresponding to dark and light areas (see arrows in plot a).

The occurrence of structural modifications in the carbon gels upon the incorporation of the conductive additive, and the spatial arrangement of the carbon black within the carbon gel matrix were investigated by Raman spectroscopy and Confocal Raman imaging [27]. The Raman spectra of the pristine carbon gel and the CB are shown in Figure 2. As seen, both samples displayed the characteristic broad D and G bands of carbon materials between 1000 and 2000 cm^−1^, with the intensity of the G band (assigned to ordered graphitic domains) and the contributions of the bands in the second order range of the spectrum more pronounced for the CB. This is in agreement with the disordered structure of the matrix of the carbon gels [15,20,28], compared to the order domains in the carbon black. A better indication of the spatial arrangement and distribution of the CB within the carbon gel matrix can be observed in Figure 2 for sample CPG–CB16, showing a reconstructed image corresponding to the variation of the fitted I_D_/I_G_ ratio in the scanned area. The image was reconstructed from over 300 Raman spectra in different points of the scanned area (spatial resolution of ca. 1.3 μm). For comparison, the individual Raman spectra recorded at two different points of the image (corresponding to well-differentiated zones) are presented in Figure 2 (locations are indicated with arrows). The coexistence of CB aggregates and a carbon gel matrix in the sample was observed by the color gradation. The dark zones in the reconstructed Raman map correspond to areas with low I_D_/I_G_ ratios, similar to that in the pristine CB additive. Local Raman spectrum of these dark areas (arrows) confirmed the structural order provided by the CB, with the appearance of the characteristic peak in the second order range of the spectrum. On the other hand, the light (yellow) areas represent high I_D_/I_G_ ratios, characteristic of the carbon gel matrix, as also confirmed by the local Raman spectrum. The structural order increased in the areas with a higher density of CB, with no apparent changes in the structure of the carbon/gel composites, compared to the structure of the individual components in the materials at this length scale. Similar observations were gathered by XRD patterns (Figure S2); the organic gels (before carbonization) display a broad peak between 10° and 34°, which is indicative of a completely amorphous structure in the non-carbonized gels. The sharp peaks at ca. 25° and 43° associated with the ordered graphitic structure of the carbon black are evident in the samples prepared with the additive, and their intensity increased with the amount of CB. The (002) broad reflection at ca. 22° corresponding to disordered carbons also present in the carbonized samples—due to the aromatization of the carbon network during pyrolysis.

Infrared spectroscopy analysis of the materials suggested that the CB is not chemically bounded to the gels, since the FTIR spectra of the gels before carbonization are similar regardless the amount of CB incorporated in the synthesis (Figure S3). The characteristic bands reported for these materials were obtained: C–O–C stretching of methylene ether bridges formed during the polycondensation of R/F (1213, 1092 cm^−1^); broad band between 3000–3500 cm^−1^ attributed to O-H stretching; a peak at 1720 cm^−1^ assigned to carboxylic acids, lactones, and anhydrides; the bands at 1650–1600 cm^−1^ corresponding to conjugated C=O and aromatic ring stretching; the band at 1470 cm^−1^ associated to CH_2_ bending; and a band at 880 cm^−1^ corresponding to the CH out of plane deformation in aromatic rings [17,29]. A similar conclusion about the absence of chemical bonds between the CB and the gel can be withdrawn from the carbonization yields (Table 1), which increased with the amount of CB additive in the samples. This is expected considering that the fraction of volatiles released upon carbonization—linked to the fraction of gel in the samples—is lower as the amount of CB additive increases.

### 3.3. Textural Characterization

**Figure 3:**
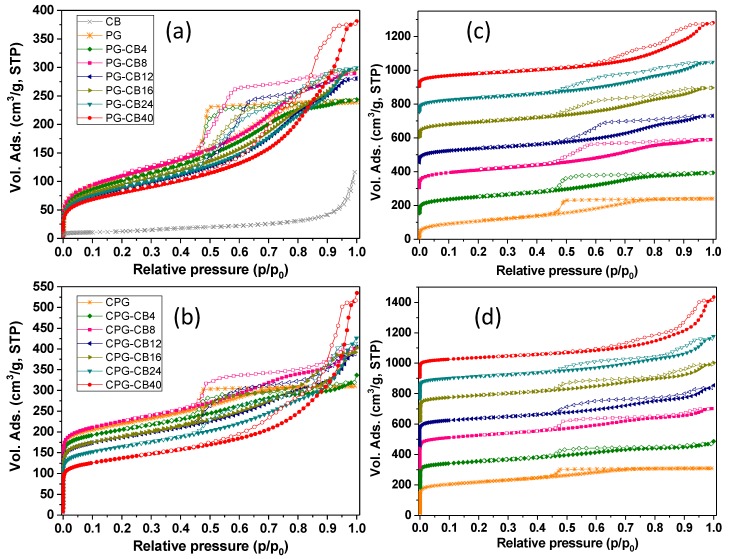
(**a**,**b**) N_2_ adsorption/desorption isotherms at −196 °C of the organic and carbon gels synthesized with different amounts of CB additive. Data corresponding to the carbon black are also shown as references. Isotherms in plots (**c**,**d**) have been shifted ca. 150 cm^3^/g for clarity.

Figure 3 shows the equilibrium nitrogen adsorption/desorption isotherms at −196 °C of all the prepared materials, including the CB additive. Important changes in the shape of the isotherms and in the amount of gas adsorbed are observed after the incorporation of the CB additive, both for the organic and the carbon gels. For the organic gels (series PG), all the isotherms displayed a type IVa character according to IUPAC (International Union of Pure and Applied Chemistry) classification [30], with a marked hysteresis loop in the desorption branch at relative pressures above 0.4. This is characteristic of materials with a well-developed microporosity and a large contribution of mesopores.

**Figure 4:**
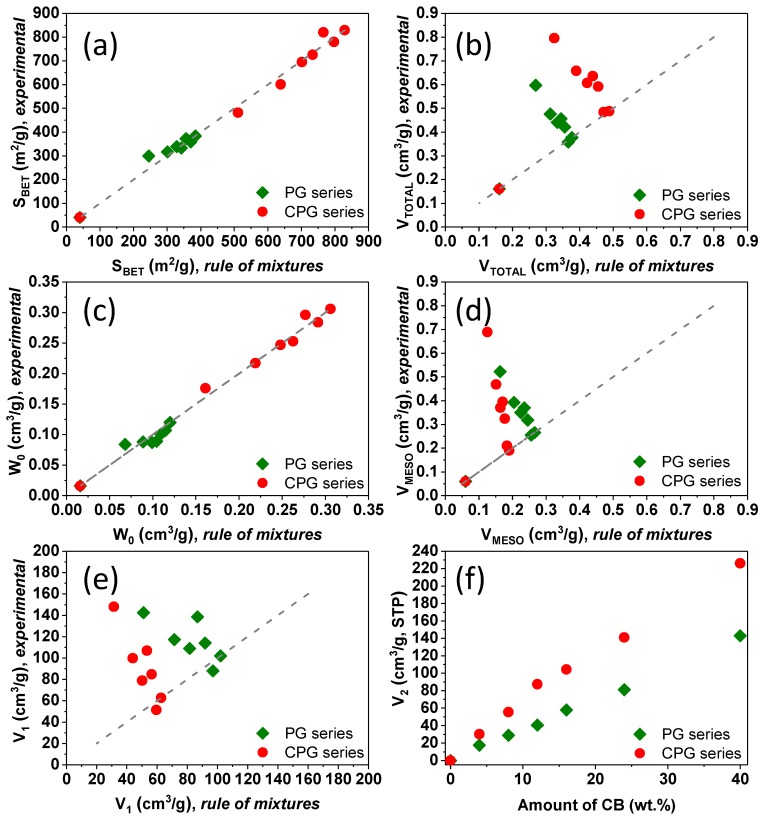
(**a**–**e**) Correlation of selected experimental textural parameters (surface area, pore volumes) of the gels/CB composites with the values predicted by the general mixing rule. Dashed lines indicate the expected trend following predictions of the general mixing rule. (**f**) Evolution of V2 with the amount of carbon additive.

As seen in Figure 3, the volume of nitrogen adsorbed increased with the amount of CB, being the effect more pronounced at relative pressures above 0.4. This indicates that the microporosity of the gels is rather unaffected by the incorporation of the CB, with a dominant impact on the mesoporosity. The values of the microporosity evaluated by the DR equation (Table 1, Figure S4) confirmed this observation. Indeed, the experimental surface areas and micropore volumes matched the values predicted by a general mixing rule (Figure 4) taking into account the composition of the materials and the textural features corresponding to the individual components: the gel and the CB. This was also corroborated by the analysis of narrow microporosity from the CO_2_ adsorption/desorption isotherms at 0 °C (Figure S5). The impact in the volume of mesopores was more pronounced, as it will be discussed below.

An important feature of the gas adsorption isotherms is the evolution of the shape and the position of the hysteresis loop (Figure 3) with the amount of conductive additive. The pristine gel (sample PG) exhibited a narrow loop (type H2) between 0.4 and 0.7 of relative pressures, in agreement with previous studies using a similar reactants molar ratio [31]. For the samples prepared in the presence of CB, the hysteresis loop broadened significantly, spanning from 0.4 up to 0.9 of relative pressures. Furthermore, these samples displayed a stepped loop, with the appearance of a curvature (inflection point) in both the adsorption and desorption branches. This feature—nicely preserved in the carbonized samples—has been reported for carbon aerogels prepared with moderate amounts of carbon black following a similar protocol, and is attributed to systems with a complex multimodal mesopore structure comprised of constricted pore necks and bodies [15,32]. Above 8 wt.% of CB, the hysteresis loops become steeper and the adsorption and desorption branches are somewhat parallel over the entire range of relative pressures.

The impact of the CB in the development of the porosity of the gels was more evident in the volume distribution of the hysteresis loop within the whole range of relative pressures. For clarity, we differentiated two regions in the loops: the first one (noted as V1) corresponds to the volume adsorbed between 0.4 and the relative pressure of the inflection point (IP) in the adsorption branch; the second one (noted as V2) corresponds to the volume adsorbed at relative pressures between IP and 1. In the case of the samples without CB additive, V1 accounts for the full loop, as V2 is not detected. Interestingly, while the increase in V2 follows a linear correlation with the amount of CB additive (for both the organic and the carbon gels), the evolution of V1 is discrete (ca. an increase between 4 and 12 wt.% of CB, and a disrupt above this value) and does not follow the expected trend considering a mixing rule (Figure 4). Both findings confirm that the appearance of a secondary mesopore network is directly connected with the presence of the carbon black additive. The relative pressure of the inflection point (higher relative pressures indicate larger pore sizes) follows a similar trend to V1 in the amount of CB (Figure S4). This indicates that the CB is responsible for the enlargement of the primary mesopore structure (higher IP and V1), and for the creation of a secondary network of mesopores of larger sizes.

Regarding the size of the mesopores, the analysis of the pore size distributions (Figure S6) of the carbonized samples showed multimodal distributions of mesopores, with the average mesopore size increasing with the amount of CB additive, in agreement with the adsorption isotherms.

### 3.4. Conductivity

**Figure 5:**
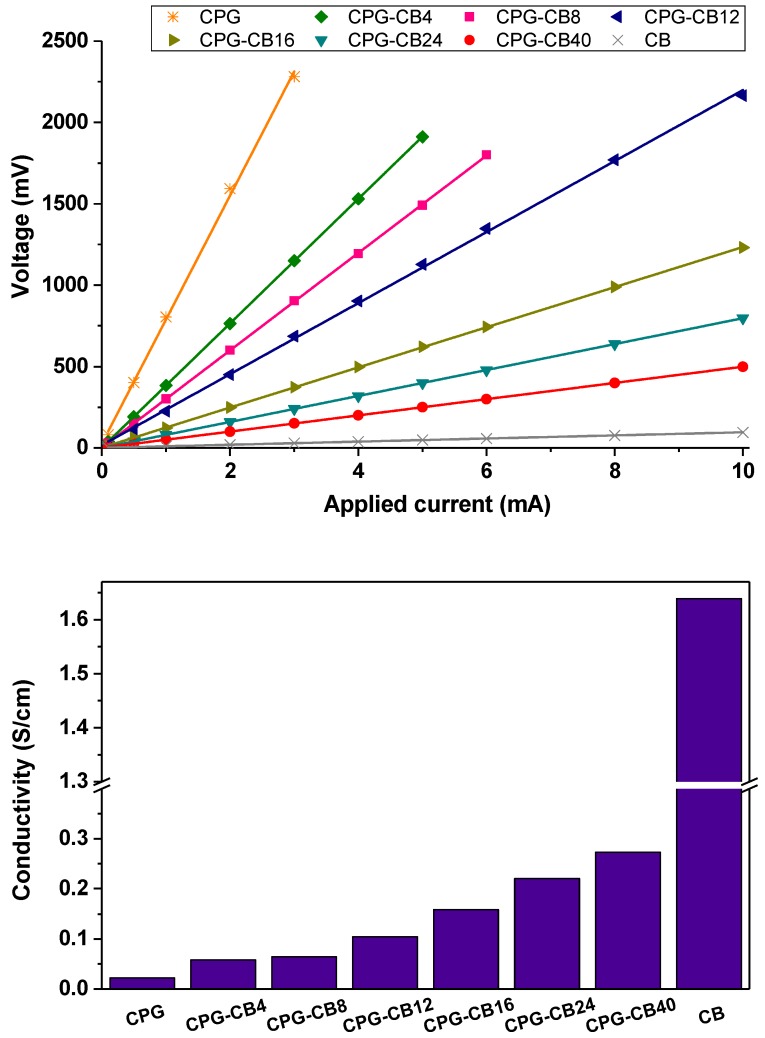
(**Top**) U–I curves and (**bottom**) electrical conductivity values for the studied carbon gel/CB composites.

Figure 5 shows the U–I (potential drop-intensity) curves obtained for pellets of the prepared materials by the four-probe measurements. The linearity of the response retrieved for all the samples confirmed that sheet resistances can be confidently evaluated obtained from the slope of the U–I curves. On the other hand, higher slopes were obtained for the materials with lower CB content, pointing to higher resistance values, and thus, lower conductivity. For instance, a decrease of ca. 12–15 times resistance was obtained for the carbon gel prepared with the highest amount of carbon black. A rise in the conductivity of the gel/CB materials was expected owing to the high intrinsic conductivity of the CB used as additive (e.g., 1.64 S/cm), compared to the poorly conductive carbon matrix of the carbon gels (e.g., 0.022 S/cm measured under the same conditions). Such a rise was, however, somewhat smaller when compared to the values reported for similar carbon electrodes when a CB additive was incorporated in the electrode’s ink (i.e., after the synthesis of the carbon material sued as electrode) [10]. This suggests a different connectivity between the CB and carbon gel particles, depending on the preparation of the electrodes.

**Figure 6:**
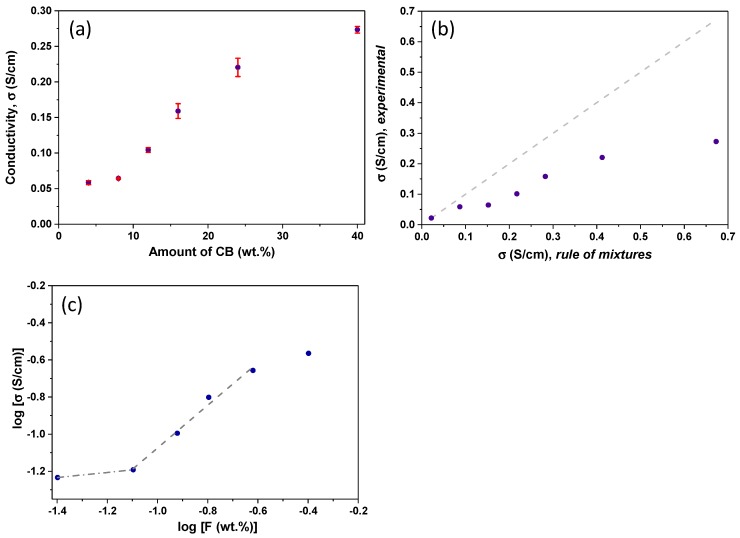
(**a**) Correlation of the electrical conductivity with the amount of carbon black; (**b**) correlation between the experimental conductivity and the values predicted by the general mixing rule; (**c**) log–log plot of the conductivity as a function of the amount of conductive phase following a percolation model.

To further clarify this aspect, the dependence of the conductivity of the gel/CB samples on the amount of conductive additive was analyzed considering a percolation model and a general mixing rule (Figure 6). The rule of mixtures would predict the conductivity of electrodes as if both components (carbon gel and CB) were segregated like a homogeneous mixture [33]. On the other hand, according to the standard percolation theory in isotropic materials, the bulk conductivity of a gel/CB composite with concentration F of a conducting phase would behave as a power law of the form [33]:

σ = σ_o_ (F − F_c_)^*β*^ (2)

where σ is the conductivity of the carbon gel/CB composite (S/cm), σ_o_ is characteristic conductivity of the carbon gel without additive (S/cm), F is the fraction of the CB additive (wt.%), F_c_ is the fraction of the additive at the percolation threshold, and *β* is a critical exponent related to the dimensionality of the material [34].

As seen in Figure 6, the experimental conductivity data of our gel/CB composites cannot be fitted to the rule of mixtures, as it did not follow a linear correlation with the amount of CB additive. Furthermore, experimental values were ca. 1.5–3 times lower than those predicted by the mixing of rules (Figure 6b), indicating that the poorly-conductive layer of the carbon gel mixed among the CB aggregates plays a dominant role in the conductivity of the bulk gel/CB composites. Conductivity values followed a power-like regime with the amount of CB additive, characteristic of percolating systems. Below 8 wt.% of CB, the conductivity of the carbon gel/CB composites was lower than expected by a mixing rule, and similar to that of the carbon gel without additive. Above this value an abrupt change is observed, suggesting a different regime governing the electronic transport properties of the composites (even though the conductivity is still lower than the value predicted by the mixing rules, and that of the CB particles alone). This can be attributed to the presence of a 3D electrically conductive network provided by the CB particles within the carbon gel matrix, which would facilitate the electron mobility between the conductive additive particles. The percolation threshold for the conductive additive loading was evaluated by plotting the log (σ) versus log (F) as depicted in Figure 6c. The percolation threshold was found at 8–12 wt.%, where the conductivity of the gel/CB materials showed a marked increase, seen by the intersection of both straight line fits. This value is higher than percolation thresholds reported for CB and other carbon additives; for instance, 0.19–4 vol.%. for MWCNT and graphene-derived materials [10,35,36,37], 2–3 wt.% for polystyrene/graphite and epoxy/graphite composites [9,38,39], and 7.5 wt.% for carbon fiber/polyethylene composites [39,40]. This is a consequence of the synthetic route, since the CB was incorporated in the reactant’s mixture (before formation of sol-gel and the carbonization) and not in the formulation of the electrodes ink (e.g., post-synthesis of the carbon material), as is usually the case in the literature.

The two well-defined regimes corresponding to different exponent β values (i.e., intersection of lines) observed in the plot (Figure 6c), indicate that our systems follow a tunneling-percolating regime, rather than a pure percolation model [41,42,43]. In this case, the conductivity of the composites depends on tunneling processes occurring between the conducting particles of the CB; since these are embedded in a less conductive medium—the matrix of the carbon gel—the distribution function of the conducting particles within the bulk material becomes more important than the bulk composition itself. The existence of percolation-tunneling systems has been proposed for other specific distributions of conducting and insulating phases involving carbon black additives [44].

## 4. Conclusions

We have prepared a series of porous gel/carbon black composites with enhanced electrical conductivity by the incorporation of the conductive additive during the early stages of the preparation of the organic gels, as opposed to the classical approach based on a post-synthetic addition in the electrodes ink. The presence of the carbon black during the polymerization of the reactants did not only modify the conductivity of the resulting carbons after carbonization of the organic gels, but also impacted the formation of the nanoporous network. Data showed that the mesopore structure of the gels is significantly developed in the presence of the CB aggregates, with the materials showing higher mesopore volumes of larger sizes, and complex multimodal mesopore size distributions. In contrast, the surface area and microporosity followed the expected trend based on the general rule of mixtures and the composition of the carbon gel/CB composites. The absence of specific interactions between the CB nanoparticles and the reactants indicates that the former would act as a porogenic agent, controlling the growth and arrangement of the resorcinol/formaldehyde clusters around the aggregates of carbon black. As a result, the primary micropore structure of the gels remains constant (as it depends on the molar ratio of reactants), while the secondary mesopore network is much more developed. The electrical conductivity of the carbon gel/CB composites increased with the amount of CB additive, following a percolation trend and indicating the good dispersion of the additive within the carbon matrix, even for the highest amounts of CB. The percolation threshold (ca. 8 wt.% of carbon black additive) was found to be higher than that reported for other additives, which is due to the different approach herein used for its incorporation in the electrode material (i.e., pre versus post-synthesis). Nonetheless, this approach allows the preparation of highly porous carbon materials with controlled mesopore architectures and enhanced electrical conductivity, facilitating the preparation of conductive carbon electrodes either in monolithic form (as-prepared) or in powder form.

## Figures and Tables

**Table 1:** Carbonization yield and main textural parameters obtained from the N_2_ adsorption isotherms for the materials synthesized with different amounts of CB additive.

Sample	S_BET_ (m^2^/g)	V_PORES_^a^ (cm^3^/g)	W_0_^b^ (cm^3^/g)	V_MICRO_^c^ (cm^3^/g)	V_MESO_^c^ (cm^3^/g)	Carbonization Yield (%)
CB	40	0.161	0.016	−	0.060	97 *
PG	384	0.377	0.120	0.091	0.266	−
PG-CB4	359	0.358	0.107	0.084	0.255	−
PG-CB8	372	0.421	0.101	0.085	0.318	−
PG-CB12	333	0.456	0.089	0.070	0.370	−
PG-CB16	339	0.441	0.087	0.074	0.350	−
PG-CB24	317	0.476	0.088	0.066	0.393	−
PG-CB40	299	0.597	0.084	0.058	0.522	−
CPG	829	0.488	0.306	0.289	0.19	51
CPG-CB4	780	0.484	0.284	0.272	0.210	52
CPG-CB8	820	0.592	0.296	0.368	0.324	53
CPG-CB12	726	0.636	0.253	0.229	0.395	55
CPG-CB16	695	0.607	0.247	0.223	0.371	56
CPG-CB24	601	0.658	0.217	0.183	0.468	60
CPG-CB40	482	0.796	0.176	0.133	0.689	67

^a^ Evaluated at p/p_0_ ~ 0.99; ^b^ evaluated by the DR method; ^c^ evaluated by the 2D-NLDFT-HS method; * yield corresponding to the carbonization of the carbon black under similar conditions, for comparative purposes.

## Supplementary Materials

The following are available online at https://www.mdpi.com/1996-1944/13/1/217/s1. Figure S1: Nitrogen adsorption/desorption isotherms at −196 °C of the gel/CB composites prepared with and without mechanical stirring. Inset: Images of the materials showing the distribution of the CB additive within the matrix. Figure S2: X-ray diffraction patterns of the organic and carbon gels. Figure S3: FTIR spectra of the polymeric gels before carbonization. Figure S4: Correlation of the amount of carbon black with the pore volumes and the relative pressures of the inflection point in the N_2_ adsorption isotherms for the polymeric and the carbon gels. Figure S5: CO_2_ adsorption isotherms at 0 °C of the gel/CB composites. Figure S6: Pore Size Distribution (PSD) of the carbon gels/CB composites evaluated from the nitrogen adsorption data and applying 2D-NLDFT-HS model.
