# Supplementary material for: Carbon Black as Conductive Additive and Structural Director of Porous Carbon Gels
Source: Materials (Basel). 2020 Jan 4;13(1):217. doi: 10.3390/ma13010217 (PMC6981367; doi:10.3390/ma13010217)
Supplement: Supplementary file 1 [file materials-13-00217-s001.pdf]

materials

MDPI

Article

Carbon black as conductive additive and structural director of porous carbon gels

Ana Casanova[1], Alicia Gomis-Berenguer [1,2], Aurelien Canizares[1], Patrick Simon[1], M. Dolores Calzada[3] and Conchi O. Ania [1,*]

[1] CEMHTI, CNRS (UPR 3079), Univ. Orléans, 45071 Orléans, France; ana.casanova-martinez@cnrs-orleans.fr; alicia.gomis@cnrs-orleans.fr; aurelien.canizares@cnrs-orleans.fr; patrick.simon@cnrs-orleans.fr; conchi.ania@cnrs-orleans.fr;

[2] ICMN, CNRS (UMR 7374), 45071 Orléans, France; alicia.gomis-berenguer@cnrs-orleans.fr (current affiliation);

[3] Laboratorio de Innovación en Plasmas (LIPs), Universidad de Córdoba, 14071 Córdoba, Spain; md.calzada@uco.es

* Correspondence: conchi.ania@cnrs-orleans.fr; Tel.: +33 (0) 238 255513

Received: date; Accepted: date; Published: date

SUPPLEMENTARY INFORMATION

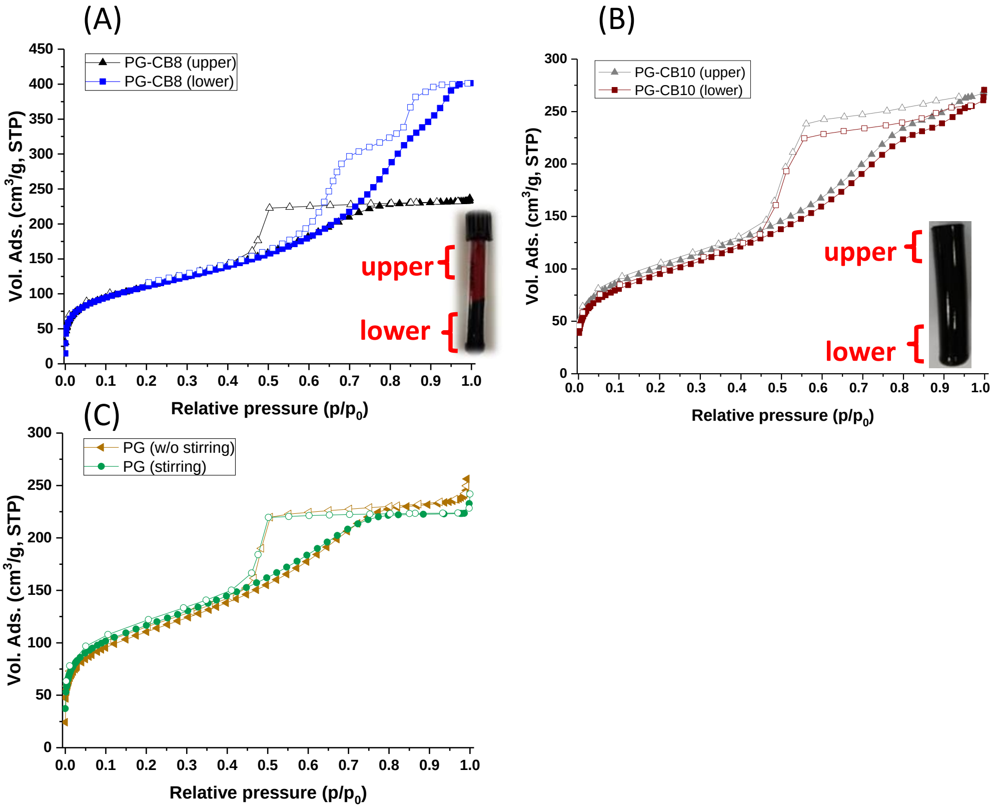

Figure S1. Nitrogen adsorption/desorption isotherms at -196 °C of a gel/CB composite (sample PG-CB8) prepared in the absence (A) and presence (B) of mechanical stirring; measurements were performed on two transversal cuts (upper and lower, see images) of the same specimen. (C) Impact of mechanical stirring of the reactants in the synthesis of the organic gel without CB additive. Inset: Images of the materials showing the distribution of the CB additive through the specimens.

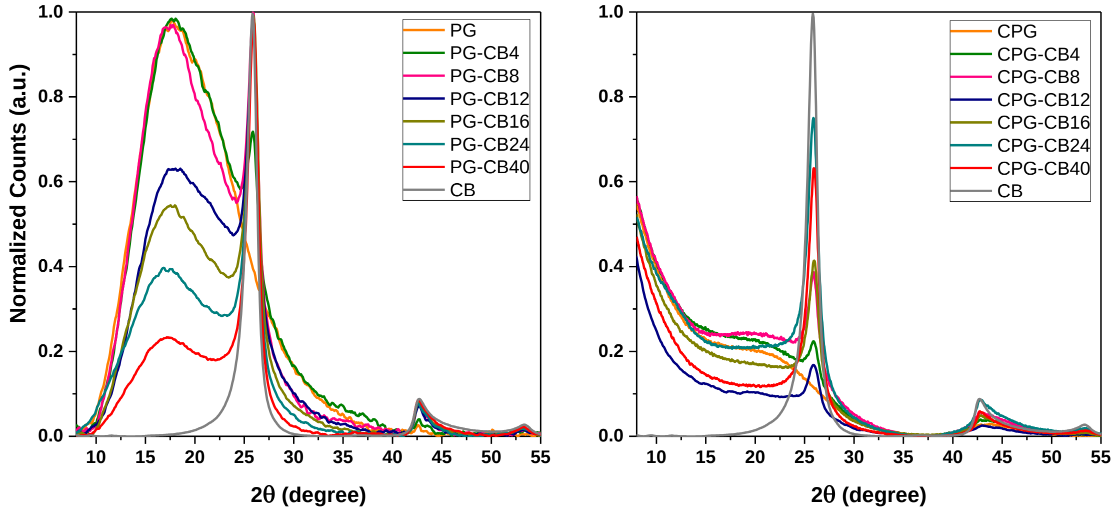

Figure S2. X-ray diffraction patterns of the organic and carbon gels.

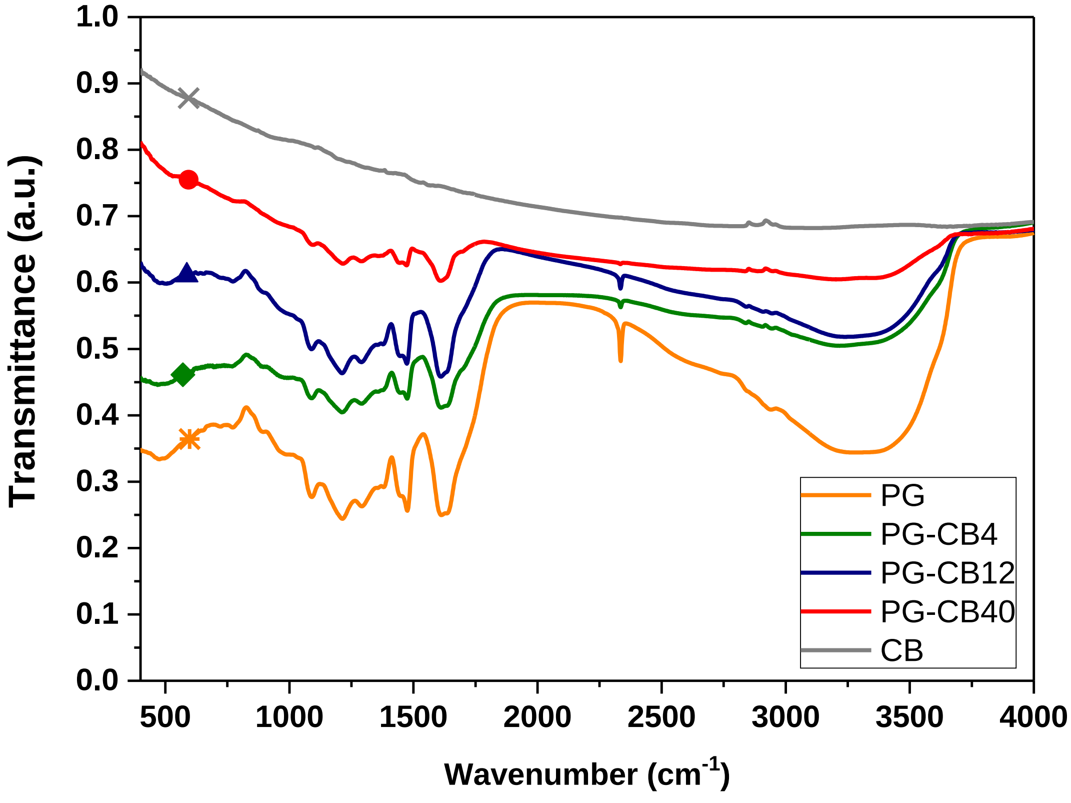

Figure S3. FTIR spectra of the organic gels before carbonization.

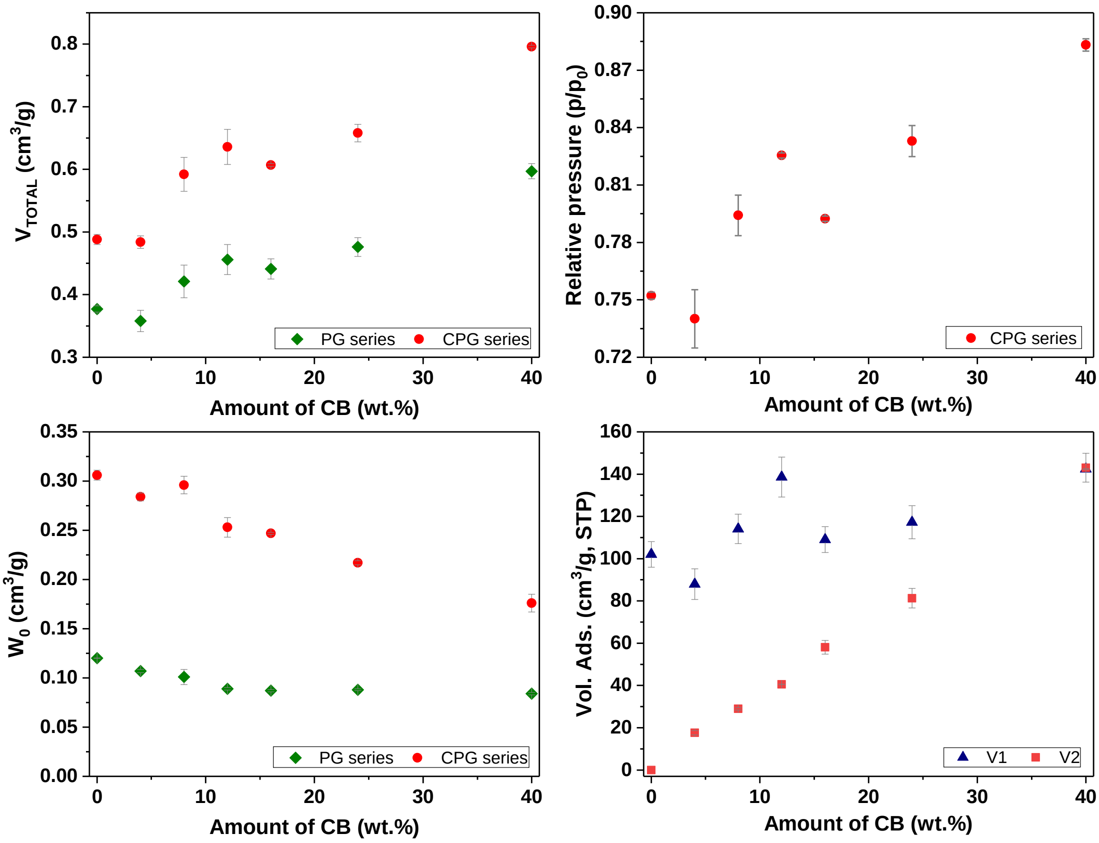

Figure S4. Correlation of pore volumes and relative pressure of the inflection point in the N_2 adsorption isotherm with the amount of carbon black.

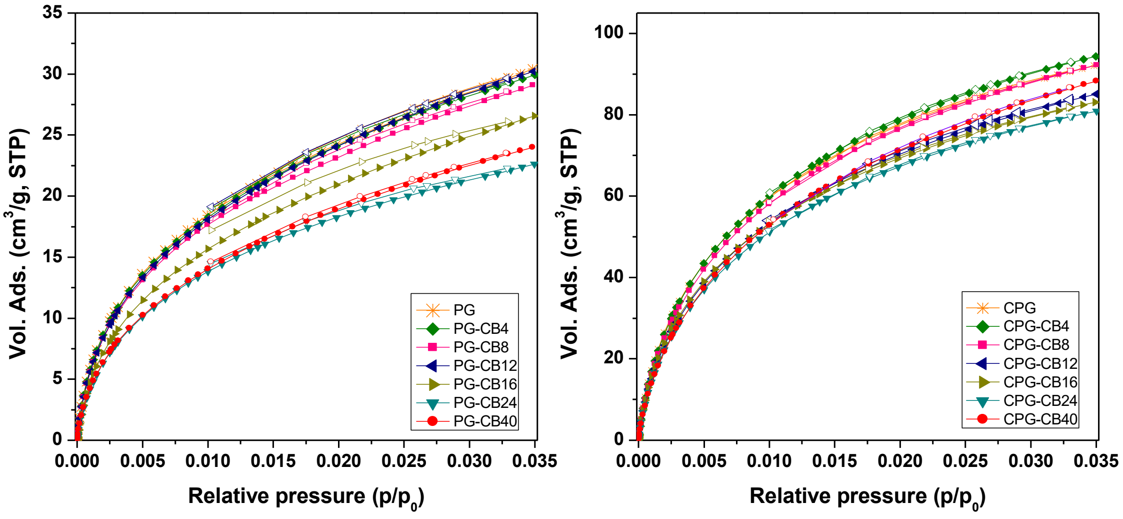

Figure S5. CO_2 adsorption isotherms at 0 ºC of the gel/CB composites.

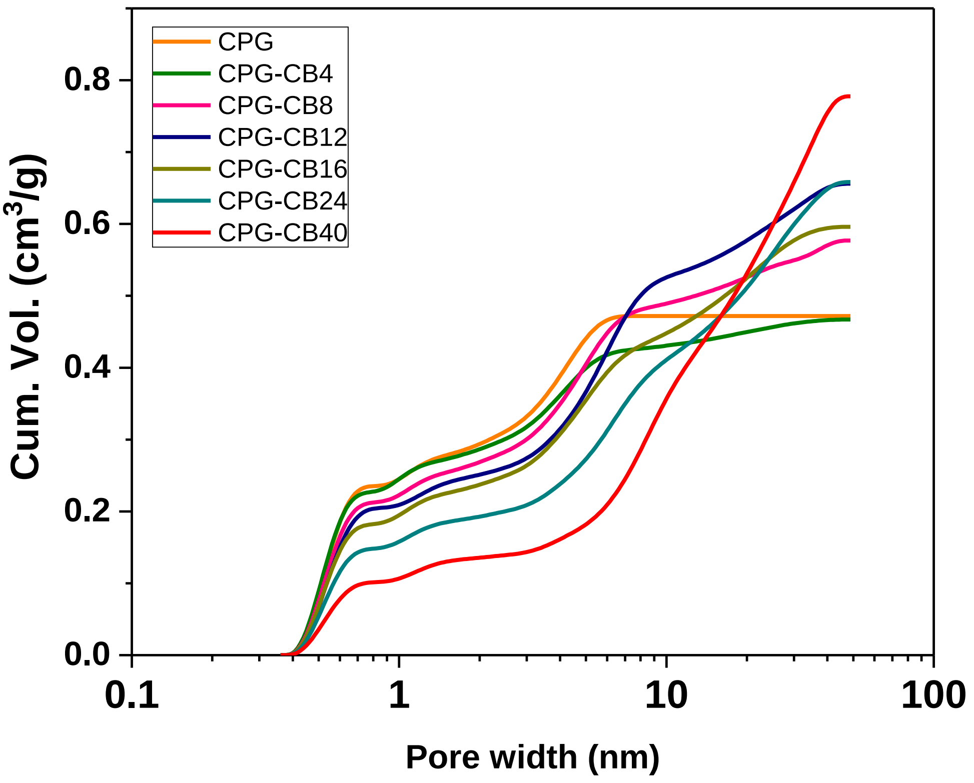

Figure S6. PSD of the carbon gels/CB composites evaluated from the nitrogen adsorption data and applying 2D-NLDFT-HS model.
